# The Mechanism of the Nucleus Accumbens–Ventral Pallidum Pathway Mediated by Drug Withdrawal-Induced High-Seeking Motivation in Cocaine Addiction

**DOI:** 10.3390/ijms252111612

**Published:** 2024-10-29

**Authors:** Jiayan Tan, Yiming Meng, Wenjie Du, Lingtong Jin, Jing Liang, Fang Shen

**Affiliations:** 1 Key Laboratory of Mental Health, Institute of Psychology, Chinese Academy of Sciences, No. 16 Lin Cui Road, Beijing 100101, China; tanjy@psych.ac.cn (J.T.); jinlt@psych.ac.cn (L.J.);; 2 Department of Psychology, University of Chinese Academy of Sciences, Beijing 100101, China

**Keywords:** cocaine, drug-seeking motivation, withdrawal, ventral pallidum, nucleus accumbens, disinhibition

## Abstract

The reinforcement of drug-seeking motivation following drug withdrawal is recognized as a significant factor contributing to relapse. The ventral pallidum (VP) plays a crucial role in encoding and translating motivational aspects of reward. However, current research lacks a clear understanding of how the VP mediates drug-seeking motivation and the feedback modulation between the VP and the nucleus accumbens (NAc) following drug withdrawal. Therefore, utilizing a rat model of cocaine self-administration, we investigated the circuitry mechanisms underlying drug-seeking behavior post-drug withdrawal involving the NAc-VP pathway. Initially, we observed a significant enhancement in drug-seeking behavior 14 days after cocaine withdrawal. Subsequently, we identified the feedback mechanism through which the NAc-VP regulates this behavior. Immunofluorescence results indicated an increase in c-Fos expression levels in the ventral pallidum ventromedial (VPvm) and ventrolateral ventral pallidum (VPvl) following drug withdrawal. Calcium fiber photometry further elucidated that during the expression of high motivational drug-seeking behavior, there was a specific enhancement in VPvm neuronal activity, and retrograde tracing techniques suggested a weakened transmission function in the NAc-VPm pathway. Additionally, chemical genetic techniques demonstrated that inhibiting the activity of the NAc-VP pathway could increase the motivational level of drug-seeking behavior. These findings indicate that the reduced inhibitory function of the NAc-VP pathway following prolonged cocaine withdrawal forms the basis for heightened reactivity in VPvm neurons, thus regulating the expression of high motivational behavior triggered by drug-related cues. Our study results suggest that maintaining normal NAc-VP pathway functionality may decrease drug-seeking motivation post long-term drug withdrawal, offering new insights for interventions targeting relapse.

## 1. Introduction

One of the central symptoms of drug addiction is relapse [1], evident in addicted individuals who are able to trigger drug-seeking or drug-using behavior even after a prolonged period of withdrawal [2]. The reinforced motivation to seek drugs post-withdrawal is identified as one of the fundamental reasons for the persistent nature of addiction [3,4].

The motivational behavior arises from the collective activity between interconnected brain regions, with the activity of these regions representing the importance of stimuli, allocating motivational value, and making appropriate decisions [2,5]. A substantial body of evidence indicates that following chronic drug exposure, drug-paired cues can significantly increase drug-seeking behavior [6] and drug-seeking motivation [7]. The behavioral task results of individuals with addiction demonstrate a decreased sensitivity to the risks associated with rewards upon attainment [8,9,10]. Furthermore, the reward value represented by drug-related cues upon re-exposure after long-term withdrawal will increase [11]. The shift in the incentive salience of drug-related stimuli and the aversive responses observed during withdrawal are intricately linked to motivational processes.

In the study of motivational behavior, the NAc is recognized as a crucial component of the dopamine (DA) neural pathway [12]. Structurally and functionally, it can be classified into distinct subregions known as the shell and core [13]. Previous research has indicated that heightened activation of NAc neurons in response to drug-associated cues following drug withdrawal is closely associated with intensified drug-seeking motivation [1]. During the early stages of withdrawal, DA levels in the ventral tegmental area (VTA)-NAc synaptic cleft are elevated [14], whereas with prolonged withdrawal, drug cessation results in a significant decrease in DA levels at the VTA-NAc synaptic cleft [15]. This suggests that the pronounced reduction in DA neurotransmitter levels at the NAc synaptic cleft following long-term drug withdrawal may serve as a prerequisite for NAc functional imbalance. However, the precise regulatory mechanisms of drug-seeking motivation associated with abnormal NAc activity during the drug withdrawal process remain to be elucidated.

The VP, functionally regulated by the NAc, encodes reward value and exhibits enhanced activity during withdrawal, potentially serving as a cue-triggered motivation mechanism [11]. Together, the NAc-VP form the ventral striatum-pallidum system, with the VP receiving GABAergic inhibitory projections from the NAc responsible for translating encoded information into behavior [13,16]. The VP can be subdivided into the ventral pallidum medial (VPvm), dorsal lateral (VPdl), and ventral lateral (VPvl) regions [17]. Research has shown that the VPvm primarily receives neural projections from the NAc shell mediating reward value preference, while the VPdl and VPvl mainly receive projections from the NAc core, contributing to drug-seeking behavior [17]. However, it remains unclear how the aberrant functional activity of the NAc during drug withdrawal regulates the VP’s projection function in reinforcing drug-seeking motivational behaviors.

The present study utilized a self-administration (SA) model of cocaine in rats and employed the breakpoint (BP) test to evaluate drug-seeking motivation. Through the integration of immunohistochemistry, in vivo fiber photometry, anterograde tracing techniques, and chemogenetics, the research investigated the regulatory mechanisms underlying the induction of heightened drug-seeking behavior in the NAc-VP pathway following prolonged drug withdrawal. Our findings indicate that during the expression of drug-seeking behavior, there is specific activation in the VPvm subregion, which is associated with a reduction in the inhibitory function of projections from the NAc shell to the VPvm. In conclusion, our study results suggest that the disinhibition process in the NAc shell-VPvm pathway following long-term withdrawal may be a neural circuit mechanism contributing to escalated drug-seeking motivation.

## 2. Results

### 2.1. The Motivation to Seek Medication During the Period of Withdrawal Exhibits an Inverted U-Shaped Curve

**Figure 1:**
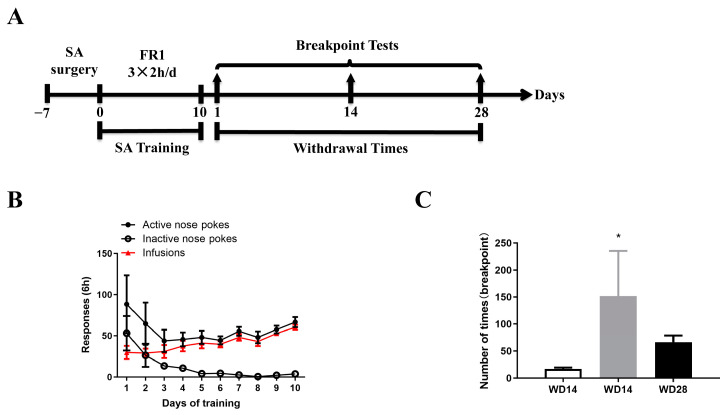
Cocaine-seeking motivation model in rats: (**A**) Behavioral protocol; (**B**) Diagram of SA behavioral training in rats (two-way ANOVA followed by Tukey’s multiple comparisons test); (**C**) Results of the drug seeking motivation test at different times of withdrawal (WD1 is the 1st day of withdrawal; WD14 is the 14th day of withdrawal; WD28 is the 28th day of withdrawal). *n* = 8 for WD1 group; *n* = 4 for WD14 group; and *n* = 6 for WD28 group. * *p* < 0.05 (WD1 vs. WD14, one-way ANOVA followed by Tukey’s multiple comparisons test).

To elucidate the changes in motivation for seeking drugs during withdrawal, we first performed jugular vein catheterization surgery to establish a cocaine SA model in rats. These rats developed cocaine dependence after SA program training and began withdrawal after 10 days. Rats were divided into three groups based on withdrawal time after SA program training, and cue-induced drug-seeking motivation levels were assessed using a BP test on Day 1 (WD1), Day 14 (WD14), and Day 28 (WD28) of withdrawal (Figure 1A). Behavioral results indicated that with increased training time, rats could effectively discriminate between active and inactive nose pokes, with a significant difference (sides: *F*_(1,220)_ = 63.38, *p* < 0.0001; days: *F*_(9,220)_ = 2.648, *p* < 0.01; Figure 1B). BP test results showed that WD14 was significantly higher than WD1 (Figure 1C), indicating that during withdrawal, cocaine-seeking motivation exhibited a reverse U-shaped trend with prolonged withdrawal time.

### 2.2. The Regional Specificity of the VP in Drug-Seeking Motivation

**Figure 2:**
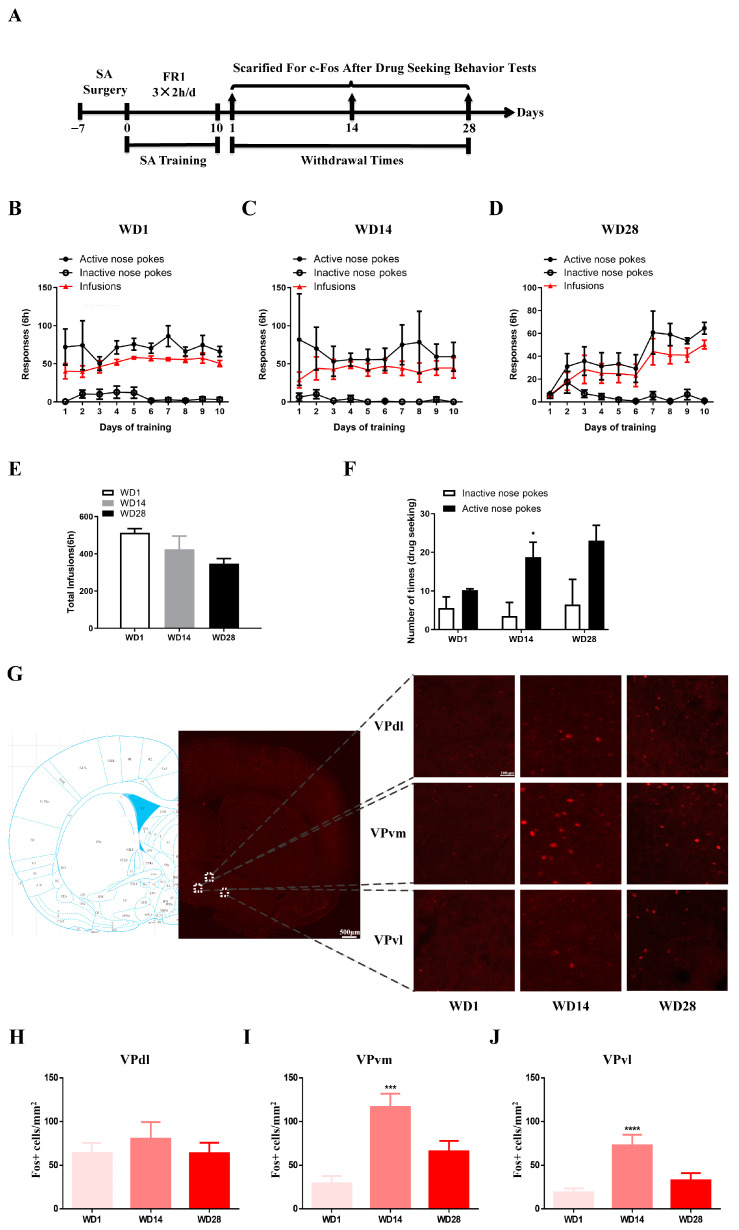
Expression of c-Fos positive cells of the VP subregions at different times during withdrawal. (**A**) Behavioral protocol; (**B**–**D**) WD1, WD14 and WD28 behavioral training diagram (two-way ANOVA followed by Tukey’s multiple comparisons test: (**B**) sides *F*_(1,80)_ = 154.189, *p* < 0.0001; (**C**) sides *F*_(1,60)_ = 45.832, *p* < 0.0001; (**D**) sides *F*_(1,80)_ = 96.565, *p* < 0.0001, days *F*_(1,60)_ = 2.341, *p* = 0.025, sides × days *F*_(9,60)_ = 3.050, *p* = 0.005); (**E**) Amount of cocaine used during the entire training period for the three groups of rats (two-way ANOVA followed by Tukey’s multiple comparisons test); (**F**) Drug-seeking behavioral test results in the three groups of rats, * *p* < 0.05; (**G**) Schematic representation of expression levels of c-Fos positive cells in VP subregions; (**H**–**J**) Statistical plots of c-Fos activation in different subregions of the VP, *** *p* < 0.001, **** *p* < 0.0001 (WD1: *n* = 5; WD14: *n* = 4; WD28: *n* = 4; one-way ANOVA followed by Tukey’s multiple comparisons test).

To clarify the role of VP and its subareas in drug-seeking motivation, behavioral procedures were conducted on rats following surgery, 10 days of SA program training, and drug-seeking behavior tests at different withdrawal times. Results from the SA program training of rats on WD1, WD14, and WD28 are presented in Figure 2B–D. Analysis of the total amount of cocaine administered during training showed no difference among the three groups (*F*_(2,9)_ = 3.173, *p* = 0.0906, Figure 2E). Subsequent drug-seeking behavior tests revealed a significant increase in active nose pokes compared to inactive nose pokes in the WD14 group, indicating that rats after 14 days of withdrawal were better able to distinguish between active and inactive sides (sides *F*_(1,16)_ = 18.174, *p* < 0.001; WD14, *p* < 0.05, Figure 2F). Following the drug-seeking behavior tests, immunofluorescent staining of c-Fos was performed on the selected rats (Figure 2G). Imaging results showed no significant effect of withdrawal time on the number of c-Fos positive cells in the VPdl (*F*_(2,89)_ = 0.4096, *p* = 0.6651; Figure 2H), but a significant effect on the number of c-Fos positive cells in VPvm and VPvl. The number of c-Fos positive cells in VPvm (*F*_(2,81)_ = 11.80, *p* < 0.001; WD1 vs. WD14, *p* < 0.001; Figure 2I) and VPvl (*F*_(2,81)_ = 10.91, *p* < 0.001; WD1 vs. WD14, *p* < 0.0001; Figure 2J) in WD14 was significantly higher than in WD1. These results suggest that the VP exhibits subregion specificity in cocaine-seeking motivation. Both VPvm and VPvl are parts of the ventral portion of the VP, while VPdl is part of the dorsal lateral VP. In terms of the functional specificity of VP subareas in cocaine-seeking behavior, it is possible that the activation levels of the ventral portion of the VP increase with longer withdrawal periods.

**Figure 3:**
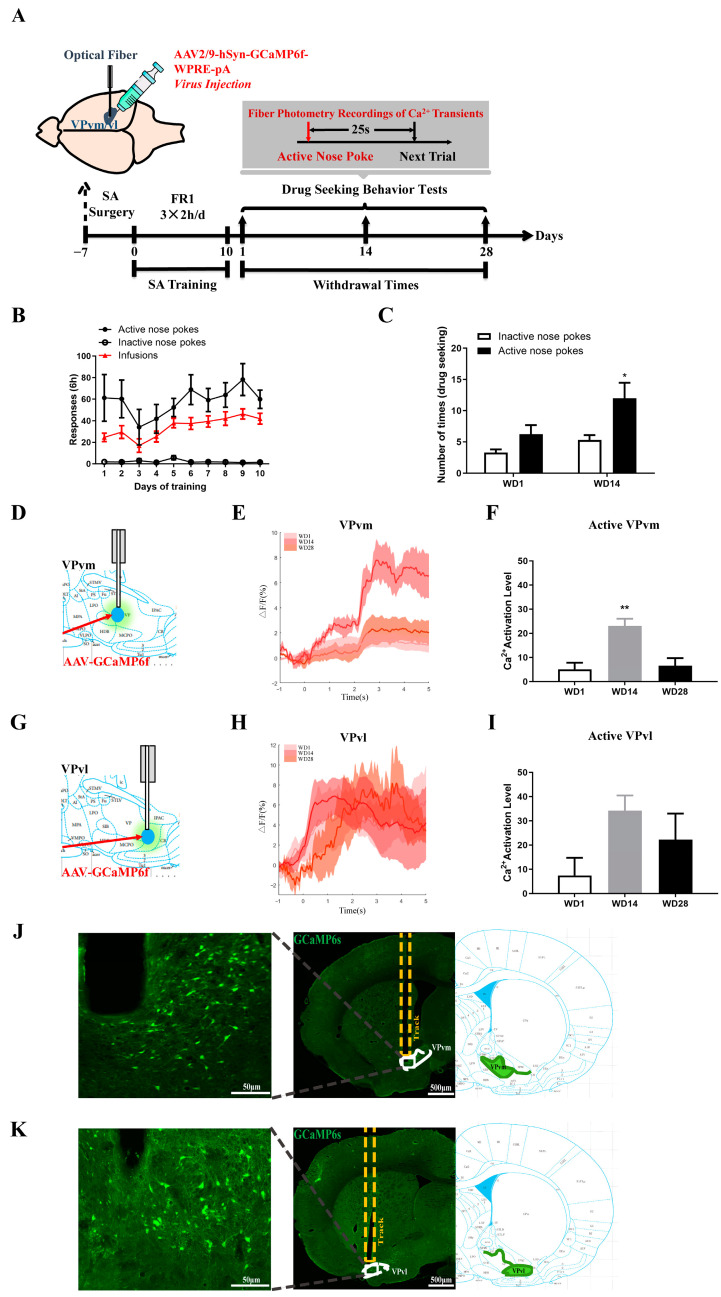
Neuronal activation in the VPvm and VPvl at different times during withdrawal. (**A**) Behavioral training and fiber photometry protocol; (**B**) Diagram of behavioral training of rats for fiber photometry (two-way ANOVA followed by Tukey’s multiple comparisons test. sides *F*_(1,220)_ = 152.8, *p* < 0.0001); (**C**) Drug-seeking behavioral test in two groups of rats, * *p* < 0.05; (**D**–**I**) Changes in neuronal calcium activity recorded in the VPvm and VPvl during the nose poking in the active side in the behavioral tests. (**D**,**G**) show positions, and (**E**,**H**) represent calcium signal changes in the VPvm during nose poking on the active side. The neuronal activation situation was characterized by calcium signaling changes within 5 s after the poking. The recorded data were calcium activity of the neuronal population, and the included data were the calcium signals in each brain side under a stable recording of calcium signal during behavioral tests. (**F**,**I**) show quantitative plots of calcium imaging results (one-way ANOVA followed by Tukey’s multiple comparisons test: (**F**) days *F*_(2,11)_ = 7.668, *p* < 0.01; ** *p* < 0.01); (**J**,**K**) Schematic diagram of the optical fiber and virus diffusion. VPvm (**J**), VPvl (**K**). (The number of animals tested was 12, and bilateral brain regions per animal, 24 calcium signal data in total, while stable recordings were included in the analysis: *n* = 10–11, * *p* < 0.05, ** *p* < 0.01, one-way ANOVA followed by Tukey’s multiple comparisons test).

However, c-Fos staining describes the activation levels of all cells in the brain regions, including neurons and glial cells. Subsequently, we further investigated the activation status of neurons in the VP subregion during the behavioral process using in vivo fiber photometry. Due to the potential impact of cocaine intake on calcium signal detection, we opted for cocaine-seeking behavioral testing (without cocaine injection, Figure 3A). Figure 3B illustrates the results of the operant conditioning training in rats, where, in the subsequent drug-seeking behavioral test, the number of active nose pokes in the WD14 group was significantly higher than the number of inactive nose pokes (sides *F*_(1,31)_ = 4.822, *p* < 0.05; groups *F*_(1,31)_ = 7.688, *p* < 0.01; Figure 3C). We injected adeno-associated viruses (AAV) expressing calcium ions bilaterally into the VPvm (Figure 3D,J) and VPvl (Figure 3G,K) and implanted optical fibers above. Taking the nose poke as time 0, we recorded changes in calcium signals from −1 to 5 s [18]. Imaging results indicated that the activation level of VPvm neurons in the WD14 group was significantly higher than in the WD1 group during active nose pokes, while there was no significant difference in the activation level of VPvl neurons. This suggests that during the behavioral testing process, the activation level of VPvm neurons increases, potentially serving as an intrinsic mechanism for drug-seeking behavior and motivation enhancement after 14 days of withdrawal (Figure 3F,I). The enhanced activation level of VPvm neurons when exposed to cocaine-related cues reflects an increase in cue reactivity, indicating that VPvm may encode cues related to reward value evaluation. These findings further elucidate the potential key role of VPvm in cocaine-seeking motivation.

### 2.3. After Withdrawal, the Neural Dominance of NAc-VP Decreases

Based on previous studies suggesting that VP receives inhibitory afferent projections from NAc [19], and that NAc-VP projections may negatively regulate motivation [20], we hypothesize that following withdrawal, the neural projections from NAc to VP diminish, mediating an increase in the activation of specific subregion neurons in VP, potentially leading to an escalation of drug-seeking motivation as withdrawal duration extends. VPvm predominantly receives neural innervation from the NAc shell. We selected the first and the fourteenth days of withdrawal as representative time points for our research on the withdrawal period.

**Figure 4:**
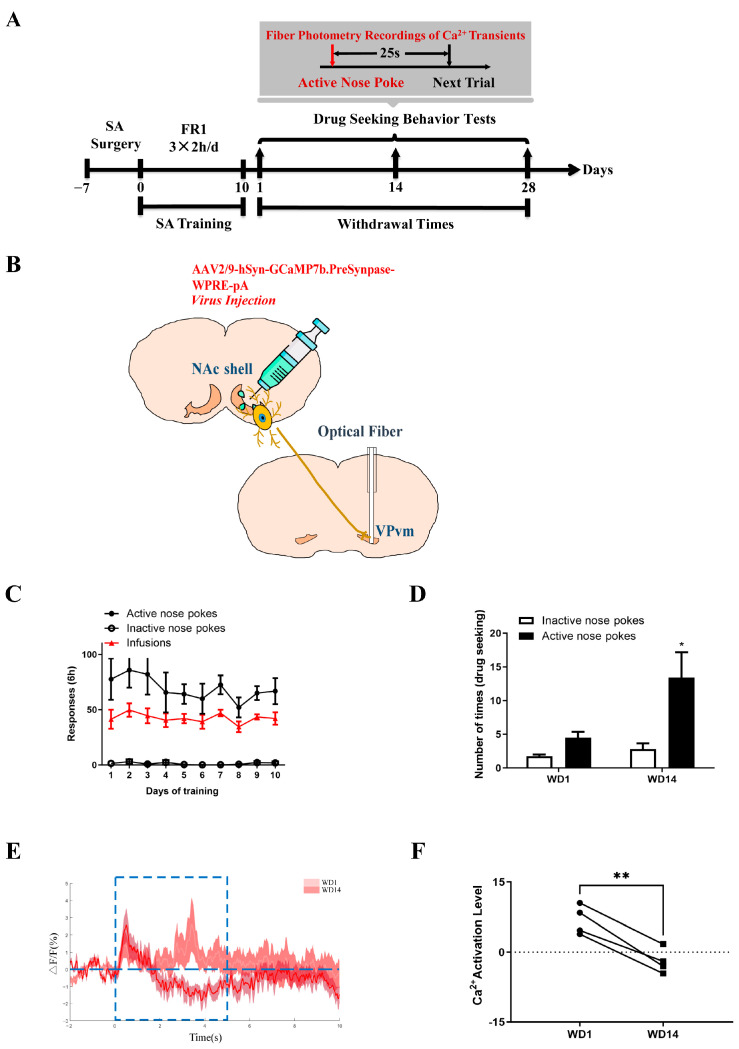
Functional alterations in neural projections from NAc shell to VPvm detected by in vivo fiber photometry. (**A**) Behavioral training protocol; (**B**) Schematic representation of virus injection and calcium fiber embedding; (**C**) Behavioral training process of the animals; (**D**) Drug-seeking behavior test during fiber photometry, * *p* < 0.05; (**E**,**F**) Neuronal activation in VPvm during the active nose poking before and after withdrawal, number of experimental animals: *n* = 8. (**E**) The dotted box shows neuronal activity 5 s after the active nose pokes. (**F**) Quantitative comparison of neuronal activity, ** *p* < 0.01.

To investigate this hypothesis, we conducted in vivo fiber photometry measurements. Figure 4A illustrates the behavioral procedure, wherein bilateral NAc shell injections of anterograde transsynaptic calcium imaging AAV were performed, with bilateral fiber implants in VPvm (Figure 4B), followed by 10 days of SA program training and drug-seeking behavior testing at different withdrawal time points. Figure 4C presents the results of the SA program training in rats. Subsequently, during the drug-seeking behavior testing, the number of nose pokes on the active side was significantly higher than on the inactive side for the WD14 group (sides *F*_(1,14)_ = 8.950, *p* < 0.01; days *F*_(1,14)_ = 4.972, *p* < 0.05; Figure 4D), indicating that rats after 14 days of withdrawal were more able to discriminate between the active and inactive sides (*p* < 0.05). Calcium imaging results revealed decreased neuronal activity in the projection projecting from NAc shell to VPvm in WD14 compared to WD1 (*t*_(5)_ = 6.438, *p* < 0.01, Figure 4E,F).

**Figure 5:**
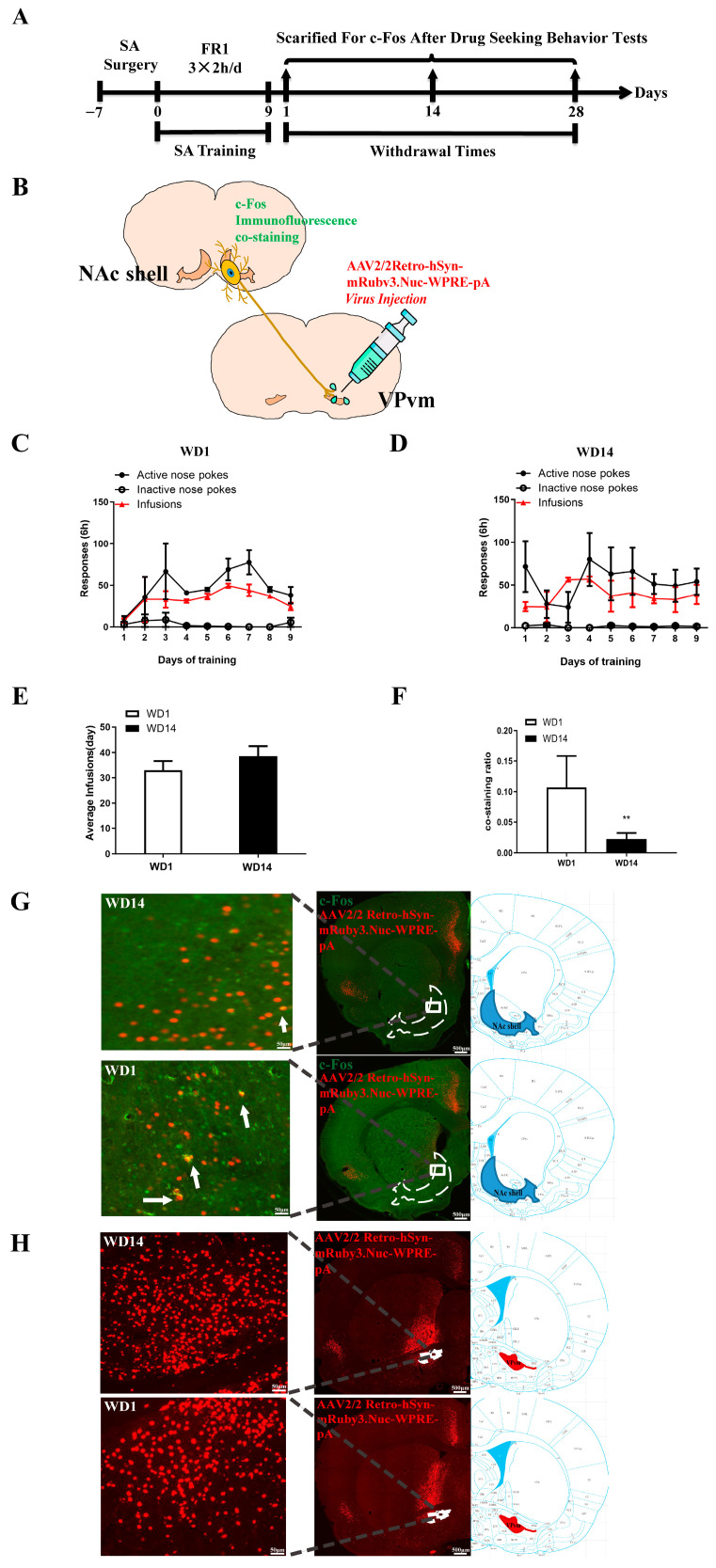
Retrograde tracing and c-Fos co-staining: (**A**) Flow diagram of experiment; (**B**) Schematic representation of retrograde tracing and c-Fos co-staining; (**C**,**D**) Behavioral training diagram of the WD1 (**C**) and WD14 (**D**) rats (two-way ANOVA followed by Tukey’s multiple comparisons test); (**E**) The total dosage of the WD1 and WD14 during the training, *t*-test; (**F**) The co-staining ratio of WD1 and WD14, *t*-test, ** *p* < 0.01; (**G**) Diagram of retrograde tracing virus in WD14 and WD1 rats, the white arrows point to the co-staining cells; (**H**) Schematic of retrograde tracing virus and c-Fos co-staining on WD14 and WD1 (WD14: *n* = 6, WD1: *n* = 10).

Due to the limitations of anterograde trans-synaptic virus, we subsequently employed a retrograde tracing technique in conjunction with c-Fos immunofluorescence for further validation. Figure 5A illustrates the behavioral procedure, in which bilateral VPvm of rats were injected with retrograde tracing AAV, followed by co-staining with NAc shell and c-Fos (Figure 5B), involving 10 days of SA procedure training and drug-seeking motivation tests at different times post-withdrawal. Figure 5C,D depict the results of SA procedure training in two groups of rats on WD1 (sides *F*_(1,18)_ = 66.64, *p* < 0.0001; Figure 5C) and WD14 (sides *F*_(1,36)_ = 45.17, *p* < 0.0001, Figure 5C), respectively. Furthermore, analysis of the total cocaine intake during training revealed no difference between the two groups (*t*_(43)_ = 0.9623, *p* = 0.3413, Figure 5E). Following drug-seeking motivation level assessment in the BP test, Figure 2G was selected for c-Fos immunofluorescence staining. Imaging results indicated a higher proportion of co-staining cells in the WD1 group compared to WD14 group (*t*_(130)_ = 2.793, *p* < 0.01, Figure 5F). These results suggest that drug-seeking motivation levels were normal prior to withdrawal (Day 1), with the number of Fos-positive cells in NAc relative to NAc shell-VPvm projecting neurons being higher compared to post-withdrawal (Day 14), indicating enhanced activation of VPvm neurons mediated by the NAc-shell post-withdrawal.

### 2.4. Inhibition of the Nucleus Accumbens on the Ventral Pallidum’s Neural Regulation, the Pharmacological Agents in WD1 Group Seek to Enhance Motivational Drive

**Figure 6:**
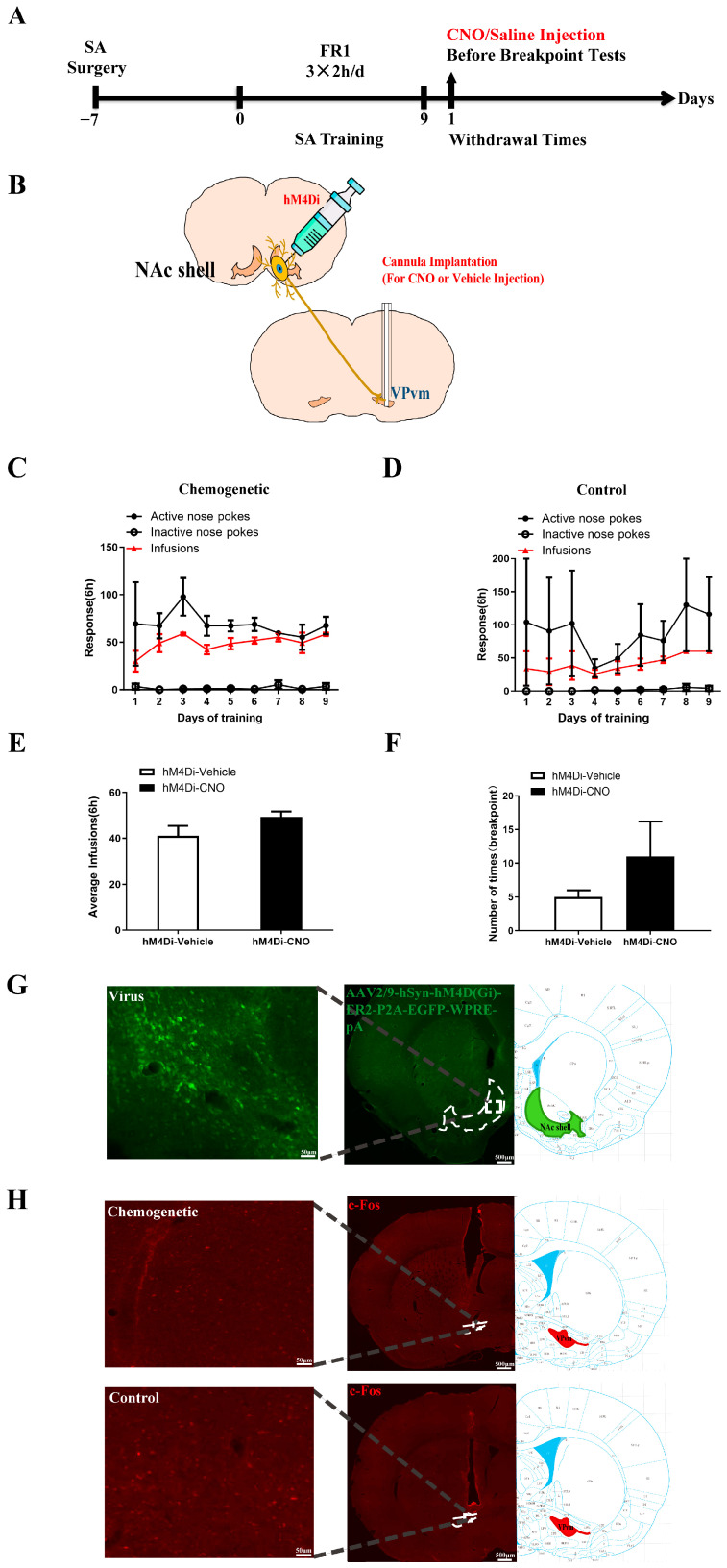
Chemogenetic inhibition of NAc to VP in drug-seeking motivation: (**A**) Flow diagram of experiment; (**B**) Schematic representation of chemogenetic inhibition of NAc to VP; (**C**,**D**) Behavioral training diagram of the chemogenetic inhibition animals (**C**) and the control animals (**D**) (two-way ANOVA followed by Tukey’s multiple comparisons test); (**E**) The total dosage of the two group animals during the training, *t*-test; (**F**) Drug-seeking motivation test, *t*-test; (**G**) Chemogenetic virus expression map; (**H**) Chemogenetic virus effect verified by the expression level of the c-Fos positive cells (up: chemogenetic inhibition, hM4Di-CNO, *n* = 6; down: control, hM4Di-Vehicle, *n* = 4).

We subsequently manipulated the NAc shell-VPvm pathway via chemical genetics to assess changes in drug-seeking motivation. Figure 6A illustrates the experimental procedure, where rats received bilateral NAc shell injections of AAV expressing hM4Di (Figure 6B), with cannulas implanted bilaterally in the VPvm for a 10-day SA training period. Preceding the withdrawal phase, animals were injected with Clozapine *N*-oxide dihydrochloride (CNO) or saline in the brain region 10 min prior to the drug-seeking motivation test. Figure 6C,D depict the results of the SA training in the CNO group (sides *F*_(1,54)_ = 121.7, *p* < 0.0001, Figure 6C) and the control group (sides *F*_(1,18)_ = 17.48, *p* < 0.001, Figure 6D). Additionally, an analysis of the total amount of cocaine obtained through training showed no difference between the two groups (*t*_(52)_ = 1.771, *p* = 0.0824, Figure 6E). BP testing for drug-seeking motivation levels revealed that animals in the CNO group exhibited a stronger motivation compared to those in the saline group (*t*_(17)_ = 0.8949, *p* = 0.3834, Figure 6F). Sections G-H of Figure 6 were selected for c-Fos immunofluorescence staining. Imaging results indicated successful inhibition of the NAc-VPvm projection by CNO, suggesting that selectively inhibiting the neural dominance from WD1 in the NAc shell to VPvm tends to increase cocaine-seeking motivation. Given the GABAergic neural dominance of the NAc shell on VPvm, this may elevate activation levels in downstream brain regions, thereby enhancing the reward value encoding cocaine-related cues and potentially leading to an increase in drug-seeking motivation.

## 3. Discussion

The present study was conducted using the rat SA model, utilizing the BP test as a measure of drug-seeking motivation to assess levels of drug-seeking behavior in rats at different time points following withdrawal [21]. The results of our motivation tests indicate that WD14 is significantly higher than WD1 and WD28, identifying a peak during the withdrawal period. This finding corroborates the inverted U-shaped curve of cue-induced drug-seeking behavior/motivation with prolonged withdrawal time [22].

The VP is primarily composed of GABAergic neurons, cholinergic neurons, and glutamatergic neurons [23,24,25], with GABAergic neurons representing the majority, while cholinergic and glutamatergic neurons are predominantly located in the VPvm subregion. Among the three subregions, VPvm has the highest neuron count [26], suggesting potential subregion-specific roles of the VP in behavioral regulation. Through immunohistochemistry and in vivo fiber photometry, we identified key subregions within the VP involved in motivated drug-seeking behaviors. Specifically, fiber photometry measurements revealed significant activation of VPvm neurons in the active side following cocaine withdrawal in rats, while the activity of VPvm neurons on the inactive side showed no significant changes. Previous studies have implicated the VPdl in drug-seeking and drug-taking behaviors [27,28,29]. However, our research findings indicate that there was no significant change in the expression levels of c-Fos-positive cells in the VPdl brain region after drug withdrawal compared to the control group. This lack of change may be attributed to the inherently higher number of positive cells. Importantly, our results primarily highlight the brain regions where significant differences exist during high-seeking drug behavior. Although VPvl showed an increase in the number of c-Fos-positive cells, there was a complete absence of neural activation in this subregion in fiber photometry tests. Previous studies have indicated that the VPvm exhibits heterogeneous discharge patterns, including distinct approach, distinct withdrawal, and approach-withdrawal responses. Hence, the VPvm neurons possess the ability to differentiate between approach and withdrawal behaviors (seeking drugs and drug intake) [30]. In pharmacological testing, the measure of effective nasal touches represents the incentive value of drug-related cues, namely drug-seeking behavior. This implies that rats have reward expectations for this behavior. The neural activation of the VPvm in this study suggests that this subregion of the VP may play a crucial role in encoding incentive value and reward expectations.

Our research findings further indicate that following cocaine withdrawal, the projection function of NAc shell-VPvm weakens, while the activity of VPvm neurons strengthens. Previous studies have demonstrated that activating GABA or enkephalin neurons in VP induces cocaine seeking, while activating glutamate neurons inhibits cocaine seeking [29]. More than 60% of neurons in VPvm are GABAergic neurons [31], and due to their predominant status, it is believed that manipulating neurons in the VP would affect their GABAergic projections. NAc-VP is a GABAergic projection that normally inhibits VPvm. A decrease in inhibitory projections results in increased activity of GABAergic neurons in VPvm. By combining anterograde tracing and fiber photometry methods, we substantiated the aforementioned statement, showing reduced calcium activity at the axon terminals of VPvm projecting from NAc shell, decreased projection from NAc shell to VPvm post-withdrawal, and a lower proportion of NAc shell to VPvm projection neurons co-stained with c-Fos-positive cells compared to WD1. VPvm may undergo a process of disinhibition, whereby GABAergic projections from NAc shell to VPvm weaken after withdrawal, leading to enhanced reactivity of VPvm neurons. This enhanced reactivity is believed to encode the incentive value of drug-related cues, ultimately resulting in heightened drug-seeking motivation.

The final step involves the application of chemical genetic techniques to regulate the activity of the NAc shell-VPvm pathway, examining the regulatory relationship between changes in pathway activity and behavior. Results from chemical genetic studies indicate that inhibiting the NAc shell-VPvm pathway leads to a heightened drug-seeking motivation similar to that observed during withdrawal. Numerous studies have indicated the involvement of the VP in the motor system [32,33]. However, within the limited sample size of experimental animals in this study, we cannot conclusively state that activating NAc’s dominance over VP weakens cocaine-seeking motivation or determine the regulatory function of neural dominance. Future research should investigate the impact of NAc-VP projection on cocaine-seeking motivation after longer withdrawal periods, such as WD14, to further elucidate the role of the NAc-VP projection pathway in cocaine-seeking motivation. Research has revealed a distinction from prior beliefs that the VP solely receives neural inputs from the NAc. VP neurons exhibit earlier and stronger activation of reward signals during the event process compared to NAc, suggesting potential projections from VP to NAc, with this pathway potentially preceding inputs from NAc to VP [34]. Studies on natural reward substances have identified a subset of GABAergic neurons from VP projecting back to the GABAergic neurons in the NAc core, known as arkypallidal VP (vArky) neurons [35]. These vArky neurons are capable of enhancing reward behaviors without inducing them, thus affecting only the value of rewards while leaving motivation unaffected. Many unresolved issues persist within the ventral striatum-pallidum system, indicating that the interactions between NAc and VP in drug addiction’s reward expectations and incentive values may be significantly more intricate than previously thought.

In this study, we have demonstrated that the neural pathway from NAc-VP contributes to the enhancement of drug-seeking motivation following withdrawal. This deepens our understanding of cue-induced drug-seeking motivation after withdrawal and extends prior views on the impact of the ventral striatum-pallidum system on drug-seeking behavior. Furthermore, our research also suggests that the VP may play a crucial role in incentive value and reward anticipation, a factor that has been overlooked for an extended period.

## 4. Materials and Methods

### 4.1. Experimental Animals

Male Sprague-Dawley rats were used in this study, all of which were purchased from Beijing Vital River Laboratory Animal Technology Co., Ltd., Beijing, China. They were acquired at 8 weeks of age and were used for behavioral and immunohistochemical experiments. Prior to the experiment, all rats were housed in the animal facility at the Institute of Psychology, Chinese Academy of Sciences. The light–dark cycle was set to 8:00–20:00, with a room temperature maintained between 22 and 25 °C. Food and water were available ad libitum. One week before the surgery, the rats were individually housed in stainless steel wire cages (22 cm × 25 cm × 30 cm) with daily handling for 5 min to minimize stress during the surgical procedure. The animals were food-restricted to maintain their body weight at 90–95% of their initial weight, approximately 280–320 g, one week prior to behavioral experiments. A total of 75 rats were included in this experiment, excluding those that did not meet the standards during the SA training and those with catheter blockages during the experiment. For specific distribution details, please refer to the figure legend.

### 4.2. Venous Catheterization Surgery

Venous catheterization surgery: Prior to surgery, a 1% pentobarbital sodium solution prepared with physiological saline was administered to the animals via intraperitoneal injection for anesthesia at a dosage of 3 mg/100 g. After achieving deep anesthesia, the hair on the back and anterior neck of the rats was shaved using a blade. Subsequently, a 2–3 cm incision was made on the back, where the skin and muscle were dissected with hemostatic forceps. A 0.5 cm incision was then made on the neck, and the muscle, blood vessels, and fascia were bluntly dissected with hemostatic forceps. The deep purple veins were identified, ligated at the distal end with sutures, and a “V”-shaped incision was made on the vein using ophthalmic scissors. A pre-made venous catheter (VABR1B/22, Instech Laboratories, Inc., Plymouth Meeting, PA, USA) was inserted from the back opening through the armpit to the anterior neck, with the tubing end inserted into the vein incision and securely sutured to prevent dislodgment. Finally, a polyester mesh was placed under the back skin and sutured in place. A mixture of 0.5 mL of heparin sodium (H8060, Beijing Solarbio Science & Technology Co., Ltd., Beijing, China) and penicillin (North China Pharmaceutical Group Co., Ltd., Shijiazhuang, China) was then infused via the venous catheter to prevent blood clotting and infection, followed by a one-week recovery period post-surgery.

### 4.3. SA Model and Training

On the 7th day following venous surgery, SA training was initiated for the rats. Before and after training each day, we assessed the patency of the catheter by observing blood reflux through a needle retraction, and then we injected a solution combining heparin sodium and penicillin into the catheter. If the catheter was obstructed, rats were expelled from the group. On the first day of training, the rats body weight was measured, and cocaine hydrochloride (Qinghai Pharmaceutical Group Co., Ltd., Xining, China) was prepared in 0.9% sterile physiological saline to the final concentrations (2.5 mg/mL). Prior to training, the rats were placed in the behavioral box (ENV-018MD, Med Associates, Fairfax, VT, USA) for 30 min of acclimatization. The rats were then placed in the operant conditioning chamber, with the venous catheter screw securely connected to the chamber screw, and underwent 3 sessions of 2 h each per day for 10 consecutive days of SA training.

The training was conducted using a Fixed Ratio 1 (FR1) schedule, with one side randomly designated as the active side and the other as the inactive side. During the FR schedule, when the rat nose poked the active port, the chamber light turned off, the infusion pump opened, and 0.1 mL of cocaine (0.75 mg/kg/infusion) was infused into the jugular vein over 3 s, accompanied by the light and sound cues for 5 s. A 20-s timeout period followed each drug infusion, during which nose pokes were recorded but no cues or drugs were delivered. After the timeout, the chamber light was turned back on. Whenever the rat nose poked the inactive port, no drug or cues were presented, and only the nose pokes were recorded. Throughout this process, the computer program logged the number of nose pokes on the active and inactive sides during each training session, nose pokes during the timeout period, nose pokes on the inactive side during the timeout, and the total number of cocaine infusions.

Our standard for conducting SA training is that during the training, rats can distinguish between active and inactive nose pokes, and the ratio of active nose pokes to the total nose pokes exceeded 75%; average daily number of cocaine injections exceeded 30, and the number of active nose pokes in the last 3 consecutive sessions fluctuated below 20%. Meeting the aforementioned criteria was considered as having completed the training. Additionally, active and inactive nose poking assignments were counterbalanced.

### 4.4. Withdrawal

Rats underwent withdrawal following the establishment of stable SA behavior. During the withdrawal period, rats were confined in their own cages and were deprived of cocaine exposure.

### 4.5. Drug-Seeking Motivation Testing

Rats were subjected to BP testing in the SA chamber after various withdrawal periods. The BP testing employed the PR49 program, wherein the number of nose pokes required for a drug injection was as follows: 1, 2, 4, 6, 9, 12, 16, 20, 25, 32, 40, 50, 62, 77, 95, 118… A higher BP value within a 2-h period indicated an elevated level of drug-seeking motivation.

### 4.6. Drug-Seeking Behavior Testing

Rats were subjected to drug-seeking behavior test in the SA chamber after various withdrawal periods. The same procedure as FR1 was used for testing, but cocaine was no longer administered, with the aim of eliminating the increase in psychoactivity caused by cocaine.

### 4.7. Brain Tissue Preparation

Following the completion of testing and behavioral training for 90 min (timing initiated upon removal of animals from the behavioral chamber), deep anesthesia was induced using a newly prepared 1% pentobarbital sodium solution. The thoracic cavity was opened with scissors, exposing the heart completely by cutting open the pericardium. A needle was inserted from the apex of the heart, passed through the left ventricle towards the aorta for saline infusion. The right atrium was incised, and the heart was flushed with water and infused with physiological saline for 15–20 min. Subsequently, the solution was replaced with 4% paraformaldehyde (BL539A, Beijing Labgic Technology Co., Ltd., Beijing, China) for 20–30 min. Following the procedure, the brain tissue was extracted and fixed in 4% paraformaldehyde solution for 8–12 h. The tissue was then dehydrated in a graded series of sucrose solutions (20% and 30%, Tianjin Guangfu Technology Development Co., Ltd., Tianjin, China) until the brain tissue settled at the bottom. After dehydration, the brain tissue was removed, excess sucrose solution on the surface was dried with absorbent paper, the brain was leveled at the base, embedded with embedding medium on the surface, frozen in a −80 °C freezer for 20–25 min. Subsequently, the tissue was thawed for 20 min in a cryostat set at −30 °C. For coronal sectioning (CM3050 S, Leica, Wetzlar, Germany), brain slices were cut to a thickness of 40 μm and stored in phosphate buffered solution (PBS, BL302A, Beijing Labgic Technology Co., Ltd., Beijing, China) solution at 4 °C.

### 4.8. Immunofluorescence

Brain sections were washed with PBS for 5 min, repeated 3 times. Subsequently, the sections were treated with a blocking solution (100% goat serum: 10% Triton: PBS = 1:2:7, Beyotime Biotech Inc., Shanghai, China) and incubated on a shaker at room temperature for 2 h to permeabilize cell membranes. The sections were then rinsed with PBS for 15 min, repeated for 3 times, followed by the addition of primary antibody (diluted 1:1000 in 5% goat serum, Abcam, Cambridge, MA, USA) and incubated at 4 °C for approximately 12–18 h. After the primary antibody incubation, the sections were washed with PBS for 15 min, repeated 3 times. The fluorescent secondary antibody was diluted in PBS (1:500, Invitrogen Corp., Carlsbad, CA, USA), and the brain sections were incubated in this solution at room temperature for 1.5 h in the dark. Subsequently, the sections were washed with PBS for 15 min, repeated 3 times. Finally, the brain sections were stained with DAPI dye diluted in PBS (1:1000, Beijing Solarbio Science & Technology Co., Ltd., Beijing, China) at room temperature in the dark for 15 min, followed by rinsing with PBS for 5 min, repeated for 3 times. After staining, the brain sections were mounted on glass slides using gelatin solution and left to air dry before further use.

### 4.9. Slice Scanning and Analysis of Numbers

Using a fluorescence microscope (DMI8, Leica, Wetzlar, Germany), neurons showing positive fluorescence were captured under a 10x objective with different excitation lights (GFP, RFP, and YFP) and corresponding images were saved for following counting analysis.

### 4.10. Virus Injection

Injecting rats with 1% pentobarbital sodium intraperitoneally for deep anesthesia, the rats were fixed on a stereotaxic apparatus, with ear bars inserted and adjusted to be in the same plane in terms of anterior-posterior and lateral positions, ensuring a depth error of no more than 0.05 mm. The scalp, with a diameter of 0.5 cm, was excised with scissors, followed by disinfection using iodine and alcohol, and then burning the meninges with 3% hydrogen peroxide (Shandong Lilkang Medical Technology Co., Ltd., Shandong, China) until the anterior and posterior fontanelles were clearly visible. The location for virus injection was determined based on the stereotaxic coordinates of the rat brain (version 6, 2007; Paxinos & Watson). Upon locating the designated area, the skull was drilled using an electric drill, and a glass electrode connected to the EP tube (1106) was inserted to deliver 1.5 μL of virus (titer > 2 × 10^12^ V.G./mL, Table 1, Shanghai Taitool Bioscience Co., Ltd., Shanghai, China) into the brain, starting from the surface of the skull to calculate the depth of penetration into the brain. After a 10-min wait, the virus was injected at a rate of 0.05 μL/min, administering 0.75 μL of virus into one hemisphere, followed by a 10-min interval before switching to the other hemisphere. The fiber optics or cannulas were fixed on the skull using three screws and dental cement. Postoperatively, a solution of penicillin with a concentration of 2.7 × 10^5^ units/mL was injected intraperitoneally at 0.5 mL to prevent postoperative infection. Following each rat injection, the glass electrode was carefully wiped and disinfected with alcohol, with electrode replacement occurring every 3–5 rats.

### 4.11. Fiber Optic Photometry

Venous catheterization surgery was finished first and the virus was injected with fiber optics embedded 0.05 mm above the corresponding brain region. Before the formal experiment, rats were acclimated to fiber bundle calcium imaging. Initially, the fiber bundle was secured to the exposed area of the rat’s head, allowing the rat to freely move for 5 min while the laser was turned on to acclimate to the laser state. Once the rat was acclimated to the secured fiber bundle, the fiber bundle was gently removed. The laser remained on for 30 min during the exploration process.

### 4.12. Retrograde Tracing Experiment

The jugular vein cannulation surgery and retrograde tracing virus injection were conducted as described previously. Five to seven days prior to the jugular vein cannulation surgery in rats, retrograde tracing virus was injected into the corresponding brain areas. One and fourteen days after cessation of the treatment, the rats underwent behavioral tests, followed by brain section preparation and immunofluorescent staining 90 min post-testing.

### 4.13. Chemogenetic Experiment

The jugular vein cannulation surgery and retrograde tracing virus injection were conducted as described previously. Five to seven days prior to the jugular vein cannulation surgery in rats, chemogenetic virus was injected into the corresponding brain areas and implanting cannulas (RWD Life Science, Shenzhen, China) above the brain regions. Prior to intervention, CNO (C0832, Sigma–Aldrich, St. Louis, MO, USA) was dissolved in Dimethyl Sulfoxide (DMSO, 17.141 mg/mL, Sigma–Aldrich, St. Louis, MO, USA) and then diluted in 0.9% saline solution, resulting in a concentration of 1 mg/mL. Similarly, a DMSO solution was prepared using saline solution as a vehicle control at the same concentration. Rats were injected with CNO or saline into the brain regions, returned to their housing cages, and subjected to a drug-seeking behavior test after 10 min. Brain section preparation and immunofluorescent staining was performed 90 min after the test.

### 4.14. Statistical Analysis

Statistical analyses were performed using GraphPad Prism 8.0 (San Diego, CA, USA).

For behavioral training and testing, data were analysed using paired-samples *t*-tests, unpaired-samples *t*-tests, or repeated measures ANOVA followed by Tukey’s post hoc test, and power analysis (Table S1).

For the immunohistochemistry experiments, we used ImageJ v1.8.0 (National Institutes of Health (NIH), Bethesda, MD, USA) to process the data. For in vivo fiber photometry, data analysis was conducted using MATLAB 2022b (MathWorks. Inc., Natick, MA, USA).

All data were shown as mean ± SEM and processed by GraphPad Prism 8.0.

## Figures and Tables

**Table 1:** Description of the viruses used in this study, including the types of viruses utilized.

Type of Experiment	Type of Viruses	Viruses
Fiber optic photometry experiment	Calcium probe	AAV2/9-hSyn-GCaMP6s-WPRE-pA
	Anterograde synaptic	AAV2/1-hSyn-GCaMP6f-WPRE-pA
	Retrograde synaptic	AAV2/2Retro-hSyn-mRuby3.Nuc-WPRE-pA
Chemogenetic experiment	Chemogenetic inhibition	AAV2/9-hSyn-hM4D(Gi)-ER2-P2A-EGFP-WPRE-pA

## Data Availability

All data needed to evaluate the conclusions in the paper are present in the paper and/or the Supplementary Materials.

## Supplementary Materials

The following supporting information can be downloaded at: https://www.mdpi.com/article/10.3390/ijms252111612/s1.
