# Supplementary material for: The Mechanism of the Nucleus Accumbens–Ventral Pallidum Pathway Mediated by Drug Withdrawal-Induced High-Seeking Motivation in Cocaine Addiction
Source: Int J Mol Sci. 2024 Oct 29;25(21):11612. doi: 10.3390/ijms252111612 (PMC11546546; doi:10.3390/ijms252111612)
Supplement: Supplementary file 1 [file ijms-25-11612-s001.zip › ijms-3235344-supplementary.pdf]

Supplementary tables

Table S1. Statistical analysis for Experiment. Statistical analysis for the behavioral results (SPSS GLM repeated-measures module, GLM univariate module, and independent-samples T-test). Partial Eta[2] (η_p^2) = proportion of explained variance. Cohen's d (d) = standardized effect size. Observed power: computed using alpha = 0.05. RM: repeated measures.

Figure number	F- or T-value	p-value	Effect size	Observed power
Figure 1B. SA active nose pokes<br>RM-ANOVA<br>Within-subject factors: Day, Side	Days (1-10) within-subject: $F_{9, 220}$ = 2.648<br>Sides (Active, inactive) within-subject: $F_{1, 220}$ = 63.380<br>Days × Sides interaction: $F_{9, 220}$ = 0.352	=0.06<br>< 0.001*<br>0.006*	η_p^2 = 0.55<br>η_p^2 = 0.55<br>η_p^2 = 0.014	0.943<br>1.00<br>0.176
Figure 1B. SA infusions<br>RM-ANOVA<br>Within-subject factor: Day	Days (1-10) within-subject: $F_{9,110}$ = 3.309	< 0.001*	η_p^2 = 0.213	0.978
Figure 1C. BP test number of times<br>RM-ANOVA<br>Within-subject factor: Group	Groups (WD1, WD14, WD28) within-subject: $F_{2, 15}$ = 4.145	=0.037	η_p^2 = 0.356	0.639
Figure 2B.WD1 SA active nose pokes<br>RM-ANOVA<br>Within-subject factors: Day, Side	Days (1-10) within-subject: $F_{9, 80}$ = 0.516<br>Sides (Active, inactive) within-subject: $F_{1, 80}$ = 154.189<br>Days × Sides interaction: $F_{9, 80}$ = 0.312	= 0.859<br>< 0.0001*<br>= 0.969	η_p^2 = 0.055<br>η_p^2 = 0.658<br>η_p^2 = 0.034	0.236<br>1.00<br>0.15
Figure 2B. WD1 SA infusions<br>RM-ANOVA<br>Within-subject factor: Day	Days (1-10) within-subject: $F_{9, 40}$ = 1.523	= 0.173	η_p^2 = 0.255	0.626
Figure 2C. WD14 SA active nose pokes<br>RM-ANOVA<br>Within-subject factors: Day, Side	Days (1-10) within-subject: $F_{9, 60}$ = 0.214<br>Sides (Active, inactive) within-subject: $F_{1, 60}$ = 45.832<br>Days × Sides interaction: $F_{9, 220}$ = 0.108	=0.991<br>< 0.001*<br>=0.999	η_p^2 = 0.031<br>η_p^2 = 0.433<br>η_p^2 = 0.016	0.112<br>1<br>0.079
Figure 2C. WD14 SA infusions<br>RM-ANOVA<br>Within-subject factor: Day	Days (1-10) within-subject: $F_{9, 40}$ = 0.424	= 0.914	η_p^2 = 0.213	0.978
Figure 2D. WD28 SA active nose pokes<br>RM-ANOVA<br>Within-subject factors: Day, Side	Days (1-10) within-subject: $F_{9, 60}$ = 2.341<br>Sides (Active, inactive) within-subject: $F_{1, 60}$ = 96.565<br>Days × Sides interaction: $F_{9, 60}$ = 3.050	= 0.025<br>< 0.0001*<br>= 0.005	η_p^2 = 0.26<br>η_p^2 = 0.617<br>η_p^2 = 0.314	0.872<br>1.00<br>0.953
Figure 2D. WD28 SA infusions<br>RM-ANOVA<br>Within-subject factor: Day	Days (1-10) within-subject: $F_{9, 30}$ = 2.689	= 0.02	η_p^2 = 0.447	0.877
Figure 2E. SA Total infusions<br>RM-ANOVA<br>Between-subject factor: Group	Groups (WD1, WD14, WD28) between-subject: $F_{2, 10}$ = 3.539	= 0.069	η_p^2 = 0.414	0.522
Figure 2F. Drug seeking behavior test (30 min) active nose pokes<br>RM-ANOVA<br>Between-subject factor: Group within-subject factor: Side	Groups (WD1, WD14, WD28) between-subject: $F_{2, 16}$ = 1.859<br>Sides (Active, inactive) within-subject: $F_{1, 16}$ = 18.170<br>Days × Sides interaction: $F_{2, 16}$ = 2.196	= 0.188<br>< 0.0001*<br>= 0.144	η_p^2 = 0.189<br>η_p^2 = 0.979<br>η_p^2 = 0.215	0.329<br>0.979<br>0.382

Figure 2F. Drug seeking behavior test (30 min) active nose pokes<br>Two-sample t-test	WD14(active, incative): t = 2.737, df = 8	= 0.077	d =1.7309	—
Figure 3B. SA active nose pokes<br>RM-ANOVA<br>Within-subject factors: Day, Side	Days (1-10) within-subject: $F_{9,\,220}$ = 0.736<br>Sides (Active, inactive) within-subject: $F_{1,\,220}$ = 152.801<br>Days × Sides interaction: $F_{9,\,220}$ = 0.864	= 0.767<br>< 0.0001*<br>= 0.559	η_p^2 = 0.029<br>η_p^2 = 0.410<br>η_p^2 = 0.034	0.361<br>1<br>0.425
Figure 3B. SA infusions<br>RM-ANOVA<br>Within-subject factor: Day	Days (1-10) within-subject: $F_{9,\,110}$ = 3.224	= 0.02	η_p^2 = 0.209	0.974
Figure 4C. SA active nose pokes<br>RM-ANOVA<br>Within-subject factors: Day, Side	Days (1-10) within-subject: $F_{9,140}$ = 0.714<br>Sides (Active, inactive) within-subject: $F_{1,140}$ = 216.066<br>Days × Sides interaction: $F_{9,140}$ = 0.491	= 0.696<br>< 0.0001*<br>= 0.879	η_p^2 =0.044<br>η_p^2 = 0.607<br>η_p^2 = 0.031	0.342<br>1<br>0.234
Figure 4C. SA infusions<br>RM-ANOVA<br>Within-subject factor: Day	Days (1-10) within-subject: $F_{9,\,70}$ = 0.552	= 0.831	η_p^2 = 0.066	0.249
Figure 4D. SA active nose pokes<br>ANOVA<br>between-subject factors: Group<br>Within-subject factors: Side	Groups (WD1, WD14) between-subject: $F_{1,14}$ = 4.972<br>Sides (Active, inactive) within-subject: $F_{1,14}$ = 8.950<br>Groups × Sides interaction: $F_{1,14}$ = 3.095	= 0.043<br>= 0.10<br>= 0.1	η_p^2= 0.262<br>η_p^2 = 0.390<br>η_p^2 = 0.181	0.546<br>0.794<br>0.374
Figure 4D. SA infusions<br>RM-ANOVA<br>Within-subject factor: Day	Days (1-10) within-subject: $F_{1,\,6}$ = 9.308	= 0.22	η_p^2 = 0.608	0.721
Figure 5C. WD1 SA active nose pokes<br>RM-ANOVA<br>Within-subject factors: Day, Side	Days (1-9) within-subject: $F_{8,18}$ = 1.571<br>Sides (Active, inactive) within-subject: $F_{1,18}$ = 66.438<br>Days × Sides interaction: $F_{1,18}$ = 1.148	= 0.202<br>< 0.0001<br>= 0.155	η_p^2 = 0.411<br>η_p^2 = 0.787<br>η_p^2 = 0.437	0.509<br>1<br>0.562
Figure 5C. WD1 SA infusions<br>RM-ANOVA<br>Within-subject factor: Day	Days (1-9) within-subject: $F_{8,18}$ = 1.393	= 0.315	η_p^2 = 0.553	0.333
Figure 5D. WD14 SA active nose pokes<br>RM-ANOVA<br>Within-subject factors: Day, Side	Days (1-9) within-subject: $F_{8,36}$ = 0.640<br>Sides (Active, inactive) within-subject: $F_{1,36}$ = 45.168<br>Days × Sides interaction: $F_{8,36}$ = 0.666	= 0.739<br>< 0.0001<br>= 0.718	η_p^2 = 0.125<br>η_p^2 = 0.556<br>η_p^2 = 0.129	0.249<br>1<br>0.249
Figure 5D. WD14 SA infusions<br>RM-ANOVA<br>Within-subject factor: Day	Days (1-9) within-subject: $F_{8,18}$ = 0.915	= 0.289	η_p^2 = 0.553	0.298
Figure 5D. SA average infusions (day)<br>Two-sample t-test	WD1 vs. WD14: t = 0.9623, df = 43	= 0.3413	d = 0.2307	—
Figure 6C. Chemogentic SA active nose pokes<br>RM-ANOVA<br>Within-subject factors: Day, Side	Days (1-9) within-subject: $F_{8,54}$ = 0.396<br>Sides (Active, inactive) within-subject: $F_{1,54}$ = 121.685<br>Days × Sides interaction: $F_{8,54}$ = 0.462	= 0.918<br>= 0.0001<br>= 0.877	η_p^2 = 0.55<br>η_p^2 = 0.693<br>η_p^2 = 0.064	0.169<br>1<br>0.194
Figure 6C. Chemogentic SA infusions<br>RM-ANOVA<br>Within-subject factor: Day	Days (1-9) within-subject: $F_{8,18}$ = 1.735	= 0.136	η_p^2 = 0.340	0.620
Figure 6D. Control SA active nose pokes	Days (1-10) within-subject: $F_{8,18}$ = 0.264	= 0.970	η_p^2 = 0.105	0.106

RM-ANOVA<br>Within-subject factors: Day, Side	Sides (Active, inactive) within-subject: $F_{1,18}$ = 17.479<br>Days $\times$ Sides interaction: $F_{8,18}$ = 0.238	=0.001<br><br>= 0.978	η_p^2 = 0.493<br><br>η_p^2 = 0.096	0.977<br><br>0.100
Figure 6D. Control SA <u>infusions</u><br>RM-ANOVA<br>Within-subject factor: Day	Days (1-9) within-subject: $F_{8,9}$ = 0.780	= 0.631	η_p^2 = 0.410	0.193
Figure 6D. SA average infusions (day)<br>Two-sample t-test	WD1 vs. WD14: t = 0.9623, df = 43	= 0.0824	d = 0.5113	—
